# Tumor microenvironment-derived serum markers as a new frontier of diagnostic and prognostic assessment in biliary tract cancers

**DOI:** 10.1002/ijc.34357

**Published:** 2022-12-01

**Authors:** Romain Désert, Fabio Giannone, Catherine Schuster, Thomas F. Baumert

**Affiliations:** 1 Université de Strasbourg, Inserm, Institut de Recherche sur les Maladies Virales et Hepatiques UMR_S1110, Strasbourg, France; 2 Pole Hépato-digestif, Hôpitaux Universitaires de Strasbourg, Strasbourg, France; 3 Institut hospitalo-universitaire (IHU), Université de Strasbourg, Strasbourg, France; 4 Institut Universitaire de France (IUF), Paris, France

## Editorial

Biliary tract cancers (BTC) are a large spectrum of anatomically and biologically different tumors sharing a dismal prognosis. While several therapeutic strategies have been approved for advanced disease, safety and efficacy remain unsatisfactory (1). Molecular background plays a pivotal role in these tumors and in their prognosis. Several mutations and protein expressions have been associated with tumoral behavior and progression and some of them have been established as independent prognostic factors in large retrospective cohorts. This is the case of KRAS, IDH, NTRK or IDH mutations and HER2 expression, which represent the target of some recently approved antitumoral drugs (2). These variables are, however, available only on surgical specimens or after tumor biopsy. In contrast serum markers, as CA 19-9, present a low sensibility in the diagnostic work-flow but are associated with patients’ outcome (3). Although other biomarkers have been tested, none of them is currently used in clinical practice. The development of rapid and easily accessible diagnostic and prognostic tools for patient outcome could guide therapeutic guidelines in these tumors by modifying decision-making work-flow, as proposing neoadjuvant systemic therapy in biological more aggressive tumors, even if resectable, as already established for other solid tumors such as pancreatic cancer.

In this journal issue, Christensen *et al*. collected samples from five prospective studies of Danish patients with BTC between January 2011 and May 2021, with blood sample collected before surgical resection or, in case of locally advanced or metastatic disease, before initiation of intended 1st-line or 2nd-line chemotherapy (4). In total, 269 patients with BTC and 49 patients with benign biliary tract disease (control) were included. The authors tested the diagnostic and prognostic use of seven circulating biomarkers of extracellular matrix (ECM) remodeling: pro-peptides of type III collagen (PRO-C3), VI, and XI, matrix metalloprotease degraded type III collagen and type IV collagen fragments, granzyme B degraded type IV collagen fragments, and MMP degraded and citrullinated vimentin. Other standard diagnosis markers were analyzed in the study, including CA 19-9, and compared with the ECM derived ones. Six out of seven ECM-related biomarkers were increased in BTC vs control. ECM derived markers did not outperform CA19-9 for BTC diagnosis but PRO-C3 outperformed CA19-9 at identifying intrahepatic cholangiocarcinoma (ICC) patients and showed similar predictive capacity than CA19-9 for the other cancer locations. Furthermore, logistic regression suggested independent predictive capacity of CA19-9 and PRO-C3 and a score combining both markers achieved a higher AUC than CA19-9 alone (AUC = 0.97 for the prediction of BTC). Four ECM markers (including PRO-C3) were significantly associated with shorter overall survival and remained significant in a multivariate Cox regression analysis including CA19-9. In patients with 2d line chemotherapy, a survival pattern similar to the 1st-line cohort was observed, and again, high PRO-C3 level was significantly associated with shorter survival.

The presence of a complex desmoplastic stroma is a distinctive histological feature of BTC. Tumor microenvironment consists of an intricate ecosystem made of non-tumor cells interacting with components of the ECM and soluble factors, involving the crosstalk of a wide variety of cells, including cancer-associated fibroblasts and immune cells and a constant ECM remodeling all along carcinogenesis and cancer progression. ECM remodeling promotes the release of pathology-specific turnover products, which may be used as quantitative biomarkers. The precursors of collagens consist of a large central triple helical region, with N- and C-terminal propeptides covalently linked to each end. The pro-peptides are released when the triple helical collagen molecules are incorporated into the growing collagen fibril (5). Hence, collagen propeptide fragments can be indicators of active ECM remodeling, which can be used for diagnosis or prognosis of BTC. Procollagens have been investigated as serum markers of chronic liver disease progression and PRO-C3 has been demonstrated to be highly related to fibrosis stage and disease activity across various chronic liver disease etiologies (5). The ELF test is a panel of markers that consists of 3 ECM components: PRO-C3, hyaluronic acid and tissue inhibitor of metalloproteinase-1. In a recent meta-analysis, it showed its capacity to identify patients with non-alcoholic fatty liver disease with a positive predictive value > 80% (6). It was also recently shown that the serum level of ECM derived proteins, including PRO-C3 can be used to diagnosticate infants with biliary atresia, a rare but extremely severe congenital disease characterized by bile duct obstruction (7). Finally, PRO-C3 and hyaluronic acid in the serum of patients with pancreatic cancer also showed some diagnostic and prognostic capacities (8).

Over the years, numerous serum markers were identified for BTC (Table 1 and reviewed in (9)), but most of them are preliminary and need to be re-assessed in different cohorts and patient subpopulations. In the current study, ECM derived markers showed promising results for BTC diagnosis and prognosis in particular the score combining PRO-C3 and CA 19-9. The results are strengthened by a well-defined study design, with a prospective cohort and data collection, and a large spectrum of biomarkers analyzed, separately or together, and then compared with other well-established markers. Nevertheless, as mentioned by the authors, a validation cohort is missing and the impact of some confounding factors, such liver disease status or comorbidities were not assessed. The area under the ROC curve of CA19-9 in the study for the diagnosis of ICC is also higher than the expected one, according to a meta-analysis of 31 studies (0.93 versus 0.83) (10), further supporting the need for an external validation. Moreover, these results represent a static assessment of biomarkers, without any correlation with tumour status or with disease evolution after chemotherapy or surgery. Radiological or pathological data could be collected and associated with peptide serum levels and a serial sampling over the follow-up period may broaden and enhance their prognostic significance. Further studies are encouraged to validate the role of these markers in specific subgroups of patients, as in those who can benefit from a curative resection, or in some subtypes of BTC, as ICC or gallbladder carcinoma.

## Abbreviations

BTC: biliary tract cancer
ECM: extracellular matrix
ICC: intrahepatic cholangiocarcinoma
PRO-C3: pro-peptides of type III collagen

## Figures and Tables

**Table 1:** Examples for serum biomarkers with diagnostic or prognostic capacity for BTC (reviewed by Macias et al. (9)). Abbreviations: AUROC, Area under the ROC curve; BTC, biliary tract cancer; DAMP, damage associated molecular pattern; ECM, extracellular matrix; EVs, extracellular vesicles.

Molecule type	Family	Member	Diagnosis	Prognosis	Level of proof	Analysed in Christensen et al.
			AUROC (BTC vs healthy)	Known association with patients survival		
Proteins	Standard	CA19-9	0.83	Yes	Very robust	Yes
		CEA	0.82	Yes	Very robust	No
Nucleic acid	cfDNA	Known mutations	NA	No	Preliminary	No
	miRs	Pooled	0.91	No	Preliminary	No
		miR-21	0.91	Yes	Preliminary	No
		miR-26a	0.9	Yes	Preliminary	No
		miR-150	0.76	No	Preliminary	No
		miR-192	0.8	No	Preliminary	No
Metabolites	Metabolomics	Six metabolites combination	0.9	No	Preliminary	No
Extracellular vésicules	EVs derived proteins	AMPN	0.88	No	Preliminary	No
		VNN1	0.88	No	Preliminary	No
		PIGR	0.84	No	Preliminary	No
		EpCAM+ASGPR1+CD133	0.82	No	Preliminary	No
	*1*	Total amount of EVs	0.81	No	Preliminary	No
Proteins	Cytokine	IL-6	0.87	Yes	Strong	Yes
	DAMP	S100A6	0.9	No	Preliminary	No
	Stem	DKK1	0.87	No	Preliminary	No
	Other	SSP411	0.91	No	Preliminary	No
	ECM	MMP7	0.73	No	Preliminary	No
	ECM/cytokine	Osteopontin	0.96	Yes	Preliminary	No
	ECM	PRO-C3	0.88	Yes	Preliminary	Yes
		PRO-C6	0.74	Yes	Preliminary	Yes
		PRO-C11	0.61	No	Preliminary	Yes
		C3M	0.78	Yes	Preliminary	Yes
		C4M	0.79	Yes	Preliminary	Yes
		C4G	0.58	No	Preliminary	Yes
		VICM	0.67	No	Preliminary	Yes
